# Supplementary material for: The oncoprotein DEK affects the outcome of PARP1/2 inhibition during mild replication stress
Source: PLoS One. 2019 Aug 13;14(8):e0213130. doi: 10.1371/journal.pone.0213130 (PMC6692024; doi:10.1371/journal.pone.0213130)
Supplement: S1 Table — (DOCX) [file pone.0213130.s001.docx]

**S1 Table Primer sequences for the site-directed mutagenesis of the DEK primary sequence**

| PBD2-Part1-fwd | GCCGAAATCTGCAGCAACTTGTAGCAAAGGCAGTAAAAAGGAACGG |
| --- | --- |
| PBD2-Part1-rev | CTGCCTTTGCTACAAGTTGCTGCAGATTTCGGCAATGGTTTGC |
| PBD2-Part2-fwd | GCAAAGGCAGTGCAGAACGGAACAGTTCTGGAATGGCAAGG |
| PBD2-Part2-rev | GTTCCGTTCTGCTGCACTGCCTTTGCTACAAGTTGCTGCAG |
| PBD2-Part3-fwd | CTGGAATGGCAGCGGCGGCTAAGCGAACCAAATGTCCTG |
| PBD2-Part3-rev | GGTTCGCTTAGCCGCCGCTGCCATTCCAGAACTGTTCCG |
| PBD2-Part4-fwd | GGAATGGCAGCGGCGGCTGCGGCAACCGCATGTCTTCTTGAAATTCTG |
| PBD2-Part4-rev | GGACATGCGGTTGCCGCAGCCGCCGCTGCCATTCCAGAAC |
| shDEK-Part1-fwd | CCAGTGCGAACCTCGAGGAAGTCACAATGAAACAGATTTGC |
| shDEK-Part1-rev | CATTGTGACTTCCTCGAGGTTCGCACTGGCCAGTAATTTC |
| shDEK-Part2-fwd | CTGGCAAGCGCGAATCTTGAGGAGGTCACAATGAAACAG |
| shDEK-Part2-rev | GTGACCTCCTCAAGATTCGCGCTTGCCAGTAATTTCTTTATTG |
