# Supplementary material for: The oncoprotein DEK affects the outcome of PARP1/2 inhibition during mild replication stress
Source: PLoS One. 2019 Aug 13;14(8):e0213130. doi: 10.1371/journal.pone.0213130 (PMC6692024; doi:10.1371/journal.pone.0213130)
Supplement: S1 Fig — (DOCX) [file pone.0213130.s002.docx]

**S1 Fig.**


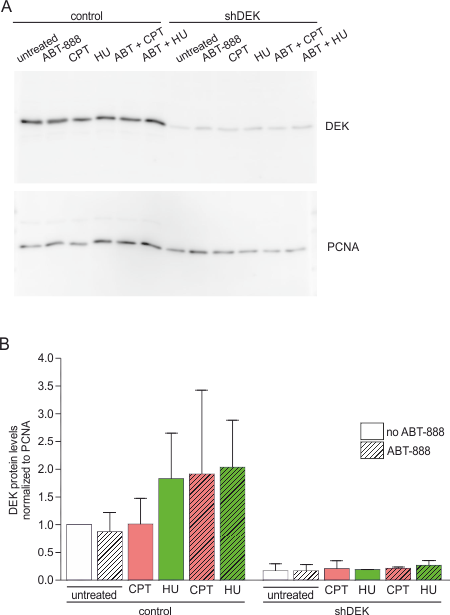


**S1 Fig. Mild replication stress does not affect DEK expression levels**

(A) Representative Western Blot of whole cell extracts from U2-OS control and shDEK cells under the replication stress conditions used in Fig.1. PCNA served as loading control. (B) Densitometric analysis. Corresponding DEK band intensities were normalized to PCNA. The data indicate the mean value of two experiments. Error bars represent the SD.
