# Supplementary material for: The oncoprotein DEK affects the outcome of PARP1/2 inhibition during mild replication stress
Source: PLoS One. 2019 Aug 13;14(8):e0213130. doi: 10.1371/journal.pone.0213130 (PMC6692024; doi:10.1371/journal.pone.0213130)
Supplement: S2 Fig — (DOCX) [file pone.0213130.s003.docx]

**S2 Fig.**


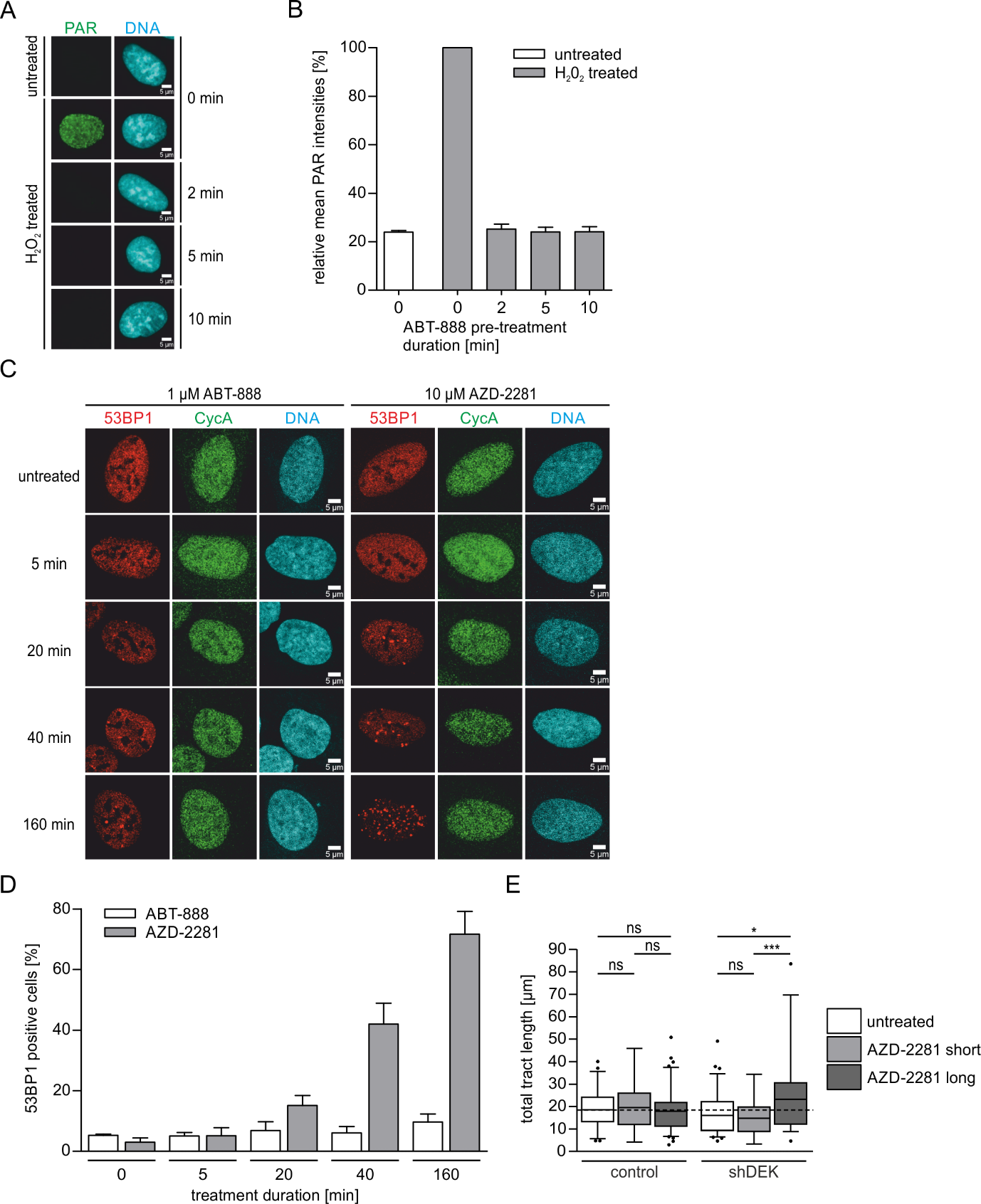


**S2 Fig. AZD-2281 induces DNA damage and accelerates fork speed**

(A-B) Inhibition of PARP1/2 activity by ABT-888. U2-OS cells were pre-treated with 1 μM ABT-888 for increasing periods of time or left untreated. PAR formation was induced with 800 μM H_2_O_2_ for 10 minutes and visualized via indirect immunofluorescence using a specific antibody (PAR-10H, green). DNA (cyan) was counterstained using Hoechst 33342. (A) Representative microscopic images are shown. (B) Quantification of PAR fluorescence intensities. Values were normalized to t=0 min after H_2_O_2_ treatment in presence of the inhibitor. At least 100 nuclei were imaged per experimental condition. The experiment was performed in triplicates. Mean values are shown. Error bars show the S.E.M. Untreated: white bar, H_2_O_2_-treated: grey bars. (C-D) Induction of DNA strand breaks in the presence of ABT-888 and AZD-2281. U2-OS cells were either treated with 1 µM ABT-888 or 10 µM AZD-2281 for increasing periods of time or left untreated. 53BP1 foci formation (red) and cyclin A expression (green) were visualized via indirect immunofluorescence. DNA (cyan) was counterstained with Hoechst 33342. (C) Representative confocal immunofluorescence images for each experimental condition are shown. (D) Quantification of results. The mean percentage of 53BP1 positive S-phase cells is shown. The amount of foci was quantified using the foci counter tool of the BIC macro tool box. At least 300 nuclei were imaged per experimental condition. The experiment was performed in triplicates. Mean values are shown. Error bars show the S.E.M. ABT-888-treated: white bars, AZD-2281-treated: grey bars. (E) DNA fiber assay: U2-OS control and shDEK cells were pulse-labelled with CldU for 20 min, followed by incubation with IdU for 20 min. 10 µM AZD-2281 was added either 2 h prior to labelling (long) or only during the ldU pulse (short). Thymidine analogues were visualized via indirect immunofluorescence. Total tract lengths of at least 39 fibers per experimental condition were scored. The bands inside the boxes display the mean, whiskers indicate the 5th to 95th percentile and black dots mark outliers. t-test: * p≤0.05, *** p≤0.001.
