# Supplementary material for: The oncoprotein DEK affects the outcome of PARP1/2 inhibition during mild replication stress
Source: PLoS One. 2019 Aug 13;14(8):e0213130. doi: 10.1371/journal.pone.0213130 (PMC6692024; doi:10.1371/journal.pone.0213130)
Supplement: S3 Fig — (DOCX) [file pone.0213130.s004.docx]

**S3 Fig.**


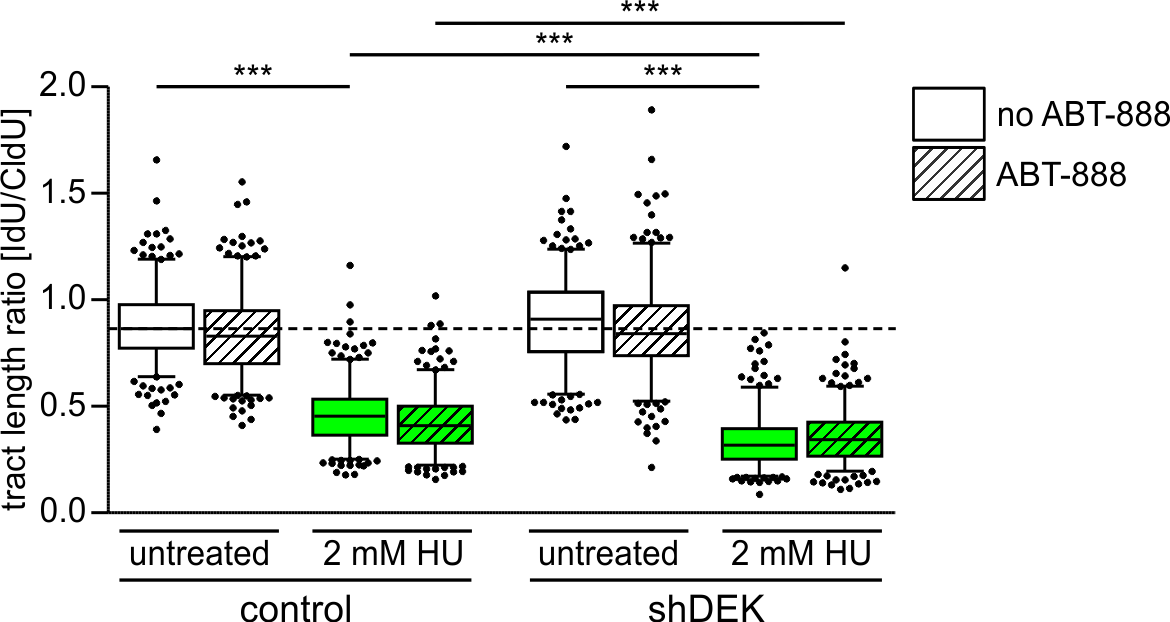


**S3 Fig. Absence of combined positive effect of DEK downregulation and PARP1/2 inhibition on fork progression under high doses of HU.**

A DNA fiber assay was performed as shown in Fig 1. U2-OS control and shDEK cells were pulse-labelled with CldU for 20 min, followed by incubation with IdU for 20 minutes in the presence or absence of 2 mM HU and 1 μM ABT-888. Thymidine analogues were visualized via indirect immunofluorescence after fiber spreading. A total of 300 tracts for each experimental condition were scored. The experiment was performed in triplicates. The bands inside the boxes display the median, whiskers indicate the 5th to 95th percentile and black dots mark outliers. t-test: *** p≤0.001. ABT-888 treated cells: hatched bars.
