# Supplementary material for: The oncoprotein DEK affects the outcome of PARP1/2 inhibition during mild replication stress
Source: PLoS One. 2019 Aug 13;14(8):e0213130. doi: 10.1371/journal.pone.0213130 (PMC6692024; doi:10.1371/journal.pone.0213130)
Supplement: S4 Fig — (DOCX) [file pone.0213130.s005.docx]

**S4 Fig.**


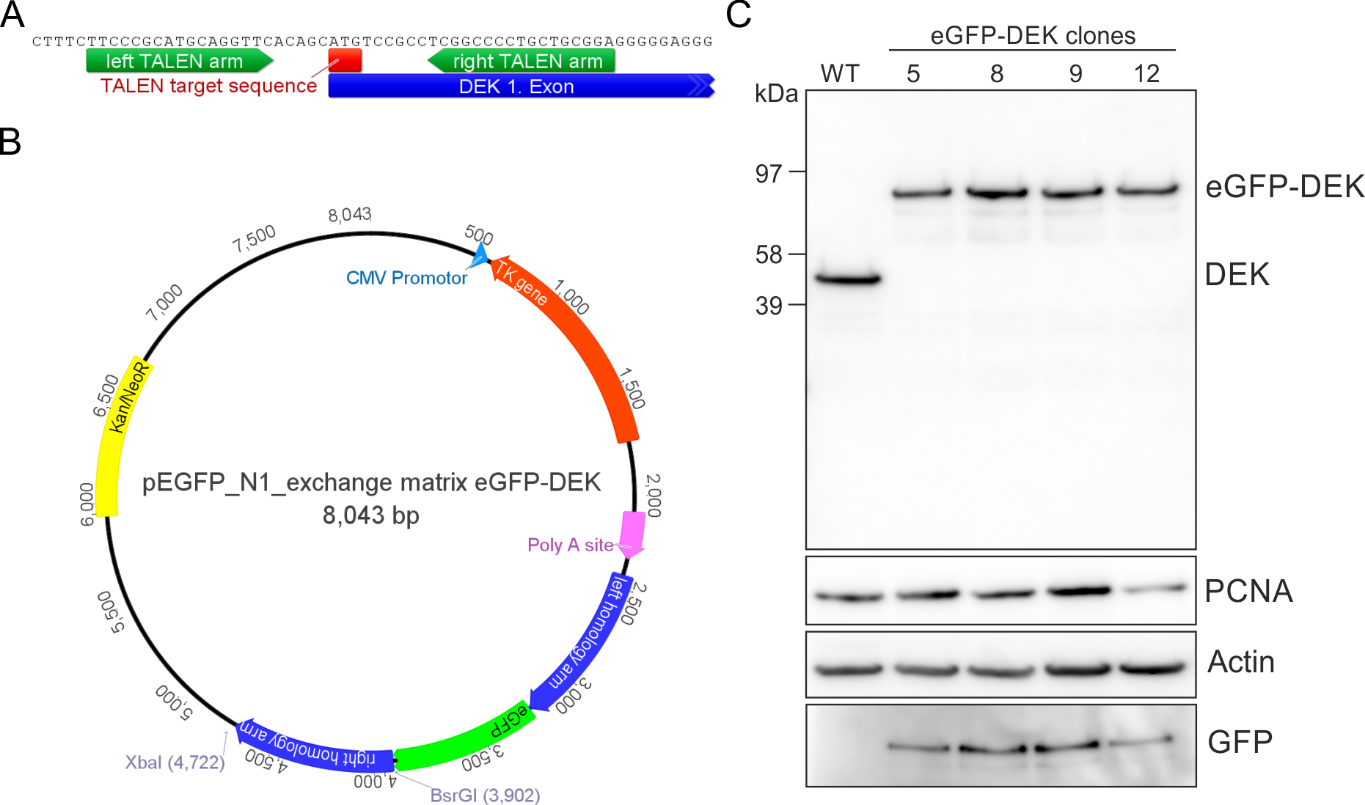


**S4 Fig. Generation of a TALEN-mediated eGFP-DEK genomic knock-in U2-OS cell line**

(A) TALENs were designed to target the start codon of the DEK gene (red). DNA binding sites of the TALEN arms are indicated in green. The first exon of the DEK gene is indicated in blue. (B) Schematic of the donor vector *pEGFP_N1_exchange matrix eGFP-DEK*. (C) Western Blot analysis of whole cell extracts from eGFP-positive clones. PCNA and actin served as loading control, GFP as a control for the successful insertion of the eGFP sequence. Clone no.8 was chosen for further experiments and named U2-OS GFP-DEK.
