# Supplementary material for: The oncoprotein DEK affects the outcome of PARP1/2 inhibition during mild replication stress
Source: PLoS One. 2019 Aug 13;14(8):e0213130. doi: 10.1371/journal.pone.0213130 (PMC6692024; doi:10.1371/journal.pone.0213130)
Supplement: S5 Fig — (DOCX) [file pone.0213130.s006.docx]

**S5 Fig.**


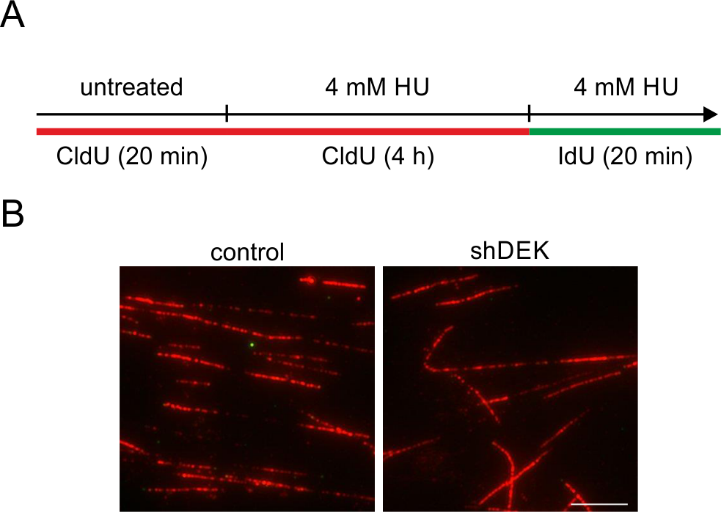


**S5 Fig. HU-induced fork arrest**

(A) Scheme of the control experiment for the fork restart assay shown in Fig 5. U2-OS control and shDEK cells were pulse-labelled with CldU (red) for 20 min, followed by incubation with 4 mM HU for four hours to arrest replication forks. Fork arrest was maintained in fresh IdU-containing (green) medium in the presence of 4 mM HU. (B) Representative images of DNA fiber spreads from U2-OS control and shDEK cells treated as in A. Scale bar: 5 μm.
