# Supplementary material for: The oncoprotein DEK affects the outcome of PARP1/2 inhibition during mild replication stress
Source: PLoS One. 2019 Aug 13;14(8):e0213130. doi: 10.1371/journal.pone.0213130 (PMC6692024; doi:10.1371/journal.pone.0213130)
Supplement: S6 Fig — (DOCX) [file pone.0213130.s007.docx]

**S6 Fig.**


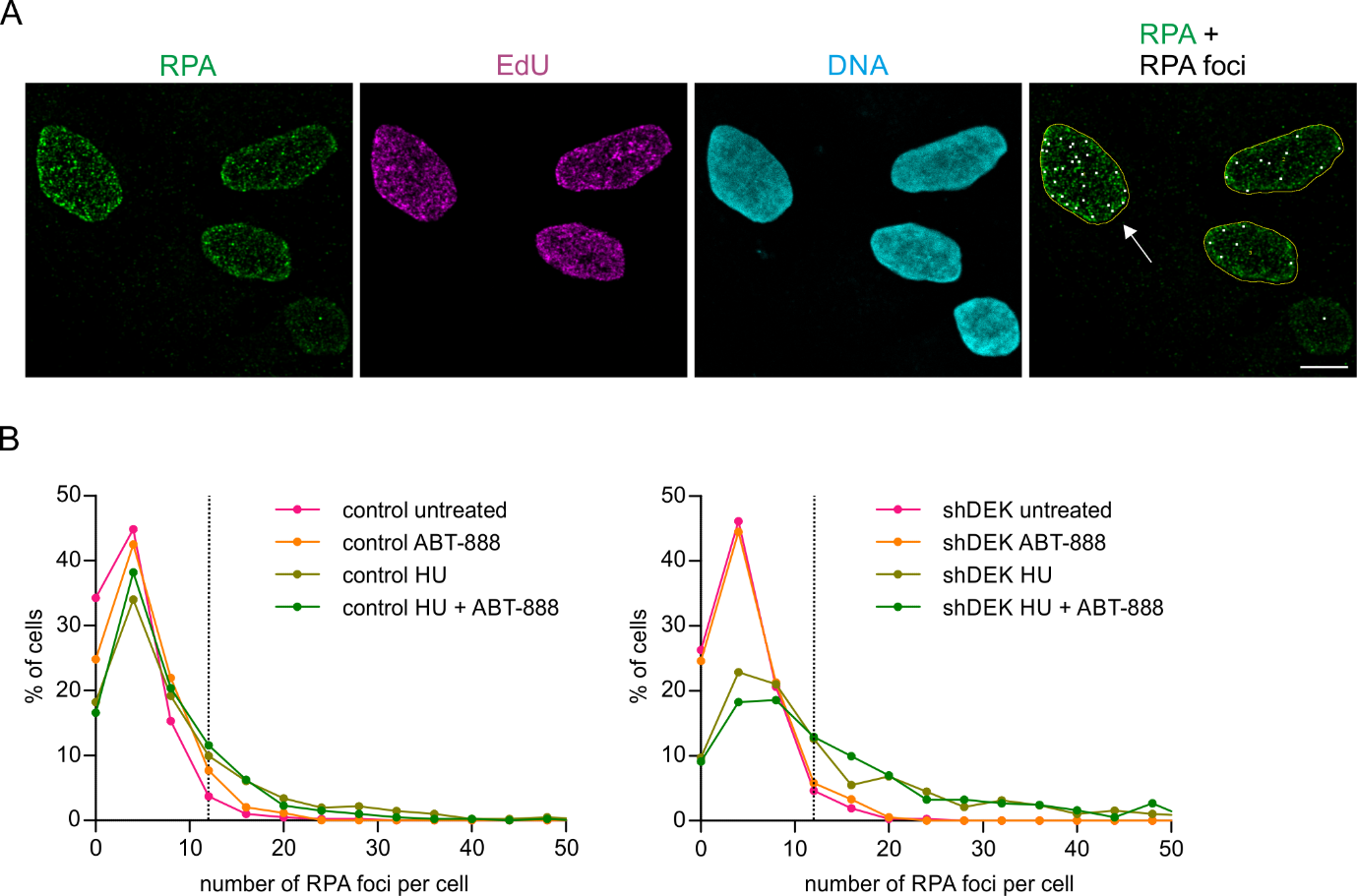


**S6 Fig. Determination of RPA-positive cells**

(A) Representative confocal image showing HU-induced RPA foci in nuclei of U2-OS cells. The experiment was performed as described in Fig 6. RPA foci were detected using the automated foci counter of the BIC macro tool box. The right image shows detected foci (white dots) superimposed on the RPA fluorescence signal. Only the left cell (white arrow) exceeded the threshold of 11 foci set for this experiment and was classified as RPA positive. Scale bar: 10 µm. (B) Histograms of RPA foci distribution in S-phase cells. Cells were treated as described in Fig 6 A. Left panel: U2-OS control cells, right panel: U2-OS shDEK cells. The dashed line marks the threshold for RPA positive cells. Bin width: 4 RPA foci per cell.
