# Supplementary material for: The oncoprotein DEK affects the outcome of PARP1/2 inhibition during mild replication stress
Source: PLoS One. 2019 Aug 13;14(8):e0213130. doi: 10.1371/journal.pone.0213130 (PMC6692024; doi:10.1371/journal.pone.0213130)
Supplement: S7 Fig — (DOCX) [file pone.0213130.s008.docx]

**S7 Fig.**


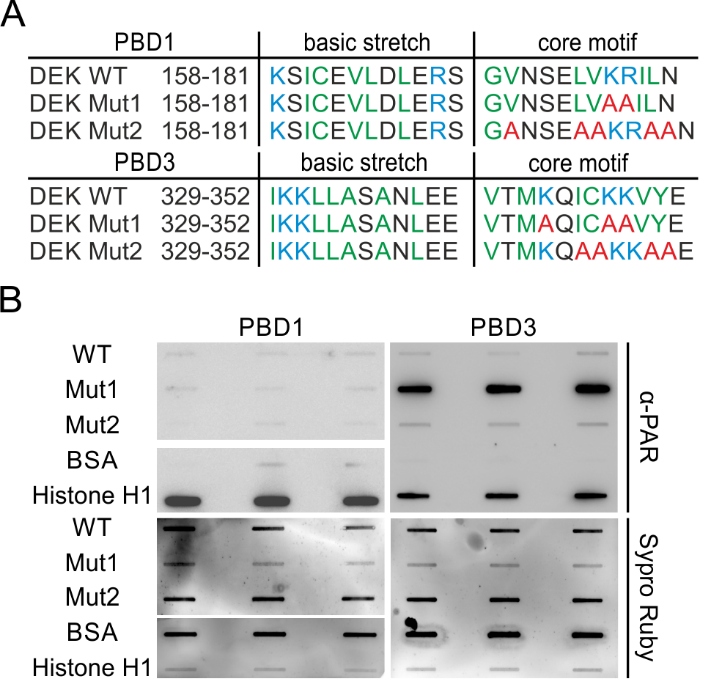


**S7 Fig. Mutational analysis of PBD1 and PBD3 using recombinant peptides**

(A) Basic (green) and/or hydrophobic (blue) amino acids were exchanged for alanine (red) as indicated. (B) Peptides were analysed in a PAR overlay assay to assess PAR-binding. PAR was detected by chemiluminescence using a specific antibody (PAR-10H). Equal membrane loading of peptides was verified using Sypro Ruby. One representative blot is shown. The experiment was performed in triplicate with similar results.
