# Supplementary material for: The oncoprotein DEK affects the outcome of PARP1/2 inhibition during mild replication stress
Source: PLoS One. 2019 Aug 13;14(8):e0213130. doi: 10.1371/journal.pone.0213130 (PMC6692024; doi:10.1371/journal.pone.0213130)
Supplement: S8 Fig — (DOCX) [file pone.0213130.s009.docx]

**S8 Fig.**


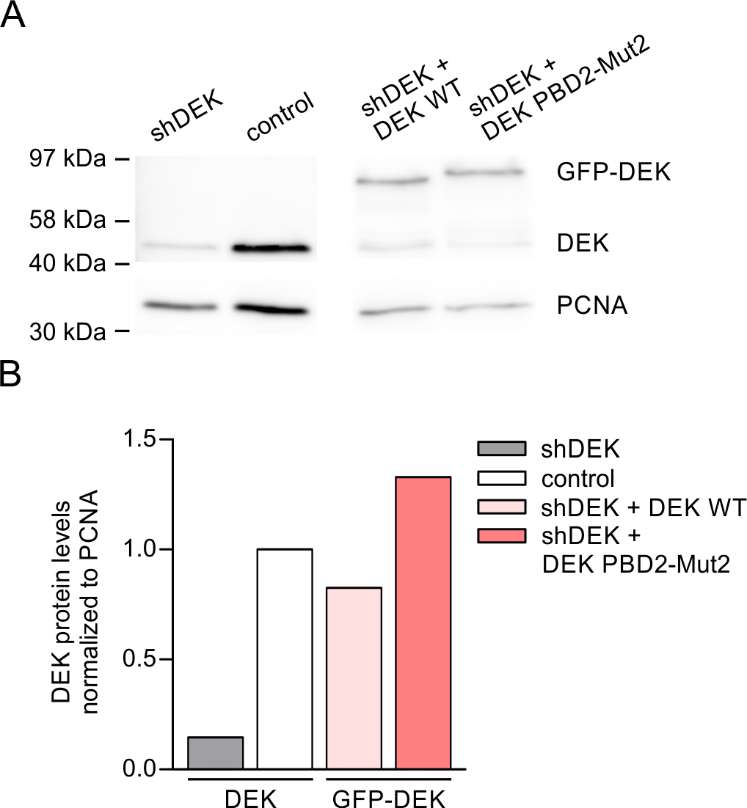


**S8 Fig. Reconstitution of U2-OS shDEK cells with DEK WT-GFP or DEK PBD2-Mut2-GFP**

U2-OS shDEK cells were transfected with plasmids encoding DEK WT-GFP or DEK PBD2-Mut2-GFP. GFP-positive, low level expressing cells were isolated using FACS. (A) Expression levels of ectopic DEK variants as well as endogenous DEK in U2-OS control and shDEK cells were visualized by Western blot. PCNA served as loading control. (B) Densitometric analysis. Corresponding DEK band intensities were normalized to PCNA and are displayed relative to endogenous DEK levels in control cells.
